# Saroglitazar ameliorates monosodium glutamate-induced obesity and associated inflammation in Wistar rats: Plausible role of NLRP3 inflammasome and NF- κB

**DOI:** 10.22038/IJBMS.2022.64041.14102

**Published:** 2022-07

**Authors:** Sayima Nabi, Uma Bhandari, Syed Ehtaishamul Haque

**Affiliations:** 1 Department of Pharmacology, School of Pharmaceutical Education & Research (SPER), Jamia Hamdard, (UGC approved deemed to be University, Govt. of India), New Delhi–110062, India

**Keywords:** Inflammation, Low-density lipoprotein – receptors, Monosodium glutamate, NLRP3 inflammasome, Nuclear factor - kappa B, Obesity, Saroglitazar

## Abstract

**Objective(s):**

Inflammation is the major progenitor of obesity and associated metabolic disorders. The current study investigated the modulatory role of saroglitazar on adipocyte dysfunction and associated inflammation in monosodium glutamate (MSG) obese Wistar rats.

**Materials and Methods:**

The molecular docking simulation studies of saroglitazar and fenofibrate were performed on the ligand-binding domain of NLRP3 and NF- κB. Under in vivo study, neonatal pups received normal saline or MSG (4 g/kg, SC) for 7 alternate days after birth. After keeping for 42 days as such, animals were divided into seven groups: Normal control; MSG control; MSG + saroglitazar (2 mg/kg); MSG + saroglitazar (4 mg/kg); saroglitazar (4 mg/kg) *per se*; MSG + fenofibrate (100 mg/kg); fenofibrate (100 mg/kg) *per se*. Drug treatments were given orally, from the 42^nd^ to 70^th^ day. On day 71, blood was collected and animals were sacrificed for isolation of liver and fat pads.

**Results:**

*In silico* study showed significant binding of saroglitazar and fenofibrate against NLRP3 and NF- κB. Saroglitazar significantly reduced body weight, body mass index, Lee’s index, fat pad weights, adiposity index, decreased serum lipids, interleukin-1β (IL-1β), tumor necrosis factor-α(TNF-α), interleukin-6 (IL-6), leptin, insulin, blood glucose, HOMA-IR values, oxidative stress in the liver and increased hepatic low-density lipoprotein receptor levels. Histopathological analysis of the liver showed decreased inflammation and vacuolization, and reduced adipocyte cell size. Immunohistochemical analysis showed suppression of NLRP3 in epididymal adipocytes and NF- κB expression in the liver.

**Conclusion:**

Saroglitazar ameliorated obesity and associated inflammation via modulation of NLRP3 inflammasome and NF- κB in MSG obese Wistar rats.

## Introduction

Obesity is a major global challenging health issue that is linked with the occurrence of several diseases such as non-alcoholic fatty liver disease (NAFLD), diabetes, and insulin resistance (1-3). 

It is a chronic metabolic disorder characterized by accumulation of fat tissue mass due to an imbalance between energy intake and energy utilization in the body (4, 5, 3). Adipose tissue functions as an immunometabolic organ and participates in the secretion of various adipokines and cytokines that regulate various physiological functions (6-8). Increased deposition of fat can lead to adipocyte cell dysfunction and dysregulated secretion of adipokines and inflammatory cytokines which, thereby, causes inflammation and insulin resistance (9, 10). Growing evidence also suggests that adipose tissue expansion is associated with low-grade inflammation and entry of various immune cells and represents the hallmark of obesity (11, 12). Adipose tissue expansion contributes to the release of various adipokines, such as leptin, insulin, proinflammatory cytokines, such as interleukin (IL)-1β, interleukin-6 (IL-6), tumor necrosis factor-α (TNF-α) in circulation, increase in expression of nuclear factor kappa B (NF-kB) activity that regulates the function of adipose tissue and various other organs in the periphery (13). Various studies have revealed the role of the NLR family pyrin domain-containing 3 (NLRP3) inflammasome in the development of adipocyte dysfunction, inflammation, oxidative stress, and insulin resistance in obesity (14, 15). The NLRP3 inflammasome, a multimeric cytosolic protein complex, releases inflammatory cytokines mainly IL-1β (16). Suppression of the NLRP3 -IL-1β pathway has been reported in the amelioration of obesity by preventing adipose tissue expansion and inflammation in various murine models (14). Therefore, this pathway is gaining much more focus to be a potential therapeutic target for treating obesity and associated inflammation.

Moreover, there is a prominent link between inflammation and altered lipoprotein metabolism resulting from dysregulated low-density lipoprotein receptors (LDLR) by inflammatory cytokines (17, 18). Peroxisome proliferator-activated receptor alpha/gamma (PPARα/γ) regulates the transcription of various genes which play a key role in regulating the metabolism of lipids, glucose, inflammation, and insulin sensitivity (19-21). PPARα plays a critical role in energy balance and regulates oxidation of fatty acids, lipid metabolism, and inflammation (22). Furthermore, PPARα activation suppresses NF-κB signaling and expression of various proinflammatory cytokines in adipose and hepatic tissue of various animal models (23, 24). Activation of PPARγ stimulates the uptake of lipids and adipogenesis, thereby enhancing insulin sensitivity (25, 26). Therefore, PPAR-γ activation, in combination with PPAR-γ, may serve as potential targets in preventing obesity, inflammation, and insulin resistance. Saroglitazar, a dual PPARα/γ agonist has been approved for management of dyslipidemia in diabetes mellitus in India after regulatory approval in 2013 (27, 28). Saroglitazar possesses a strong effect on PPAR-α receptors with a moderate effect on PPAR-γ (29). 

Previous literature has mentioned the pivotal role of monosodium glutamate (MSG) in the progression of obesity and related metabolic complications. MSG is the most commonly used flavor-enhancing additive in the food industry. High doses of MSG administration in neonatal rats lead to induction of obesity, decreased insulin sensitivity, and inflammation in adult Wistar rats (30, 31). MSG modulates the expression of various genes involved in the storage and catabolism of lipids in hepatic and adipose tissue (30). Therefore, the present study used MSG (4 g/kg, SC) to induce obesity in the Wistar rat pups. 

The prevalence of obesity continues to increase, therefore innovative treatment strategies to prevent obesity are the need of time. Various studies have shown an association of inflammatory biomarkers with obesity; therefore, an inflammatory response is a potentially modifiable risk factor (16). However, the role of saroglitazar in obesity and obesity-associated inflammation has not been explored yet. Therefore, the present study hypothesized that saroglitazar could modulate adipose tissue dysfunction, obesity-related inflammation, improve insulin sensitivity in MSG obese Wistar rats, and may prove an innovative therapeutic strategy to ameliorate obesity and associated co-morbidities. The present study investigated the antiobesity role of saroglitazar at two dose levels (2 mg/kg, PO & 4 mg/kg, PO) in MSG obese rats, and fenofibrate (100 mg/kg, PO) was used as a standard drug to compare the results.

## Materials and Methods

***Drugs and chemicals***

MSG (Analytical Grade), was obtained from Sisco Research Laboratories (SRL) Pvt. Ltd. Mumbai, India. Gift samples of saroglitazar and fenofibrate were procured from Zydus Cadila, Ahmedabad, India, and Sai Healthcare, Solan, Himachal Pradesh, India, respectively. 

***In silico studies (molecular docking)***

The molecular docking studies were performed on the ligand-binding domain of NLRP3 and NF- κB using AutoDock Vina 1.5.7. (32). The three-dimensional X-ray crystallographic structure of NLRP3 (PDB ID: 7ALV; resolution: 2.83 Å) and NF- κB (PDB ID: 1A3Q; resolution: 2.10 Å) were downloaded from RCSB Protein Data Bank (PDB). The structures of ligands, saroglitazar, and fenofibrate were downloaded from the PubChem compound database. Further, the structures of ligands were drawn in Chem Sketch and converted in pdb format using Open Babel software Version 3.1.1 (33). 

For protein preparation, the proteins were individually imported in Biovia Discovery Studio Visualizer. The bound water molecules and co-crystal ligand were deleted and polar hydrogen atoms were added. The ligands and proteins were saved in. pdbqt format in AutoDock Vina (MGL Tools 1.5.7). To establish the binding site of NLRP 3 (PDB ID: 7ALV) the grid box with dimensions 40, 40, and 40 for x, y, and z axes, respectively, and the grid center with x=14.200 Å, y=33.392 Å and z=126.100 Å having grid spacing of 0.375 Å was generated. A grid box with dimensions 126, 126, and 126 for x, y, and z axes, respectively, and a grid center with x=16.767 Å, y=61.424 Å, and z=0.663 Å having grid spacing of 0.770 Å was generated to establish the binding site of NF- κB (PDB ID: 1A3Q). The exhaustiveness in both the proteins was set to 32. The conformation in the least energy was chosen best and the dock pose was saved. The ligand interaction diagram and the dock pose were taken using the discovery studio visualizer.

***Animals***

All animal experimentations were approved by Institutional Animal Ethics Committee (IAEC) as per the Committee for the Purpose of Control and Supervision of Experiments on Animals (CPCSEA) guidelines (Approved No. 1510; Dated: 04-12-2018). Male neonatal rat pups (1 day old, 4-8 g) along with mothers were obtained from the Central Animal House Facility (CAHF) of Jamia Hamdard, New Delhi, India. Animals were maintained in normal conditions (12 hr light: dark cycle, relative humidity 60 ± 5%, and temperature 23 ± 2 °C) in polypropylene cages and had free access to a standard normal chow diet (Nav Maharashtra Chakan Oil Mills Ltd., Delhi, India) and water *ad libitum.*

***Induction of experimental obesity***

One day old male rat pups were injected MSG (4 g/kg, b.w) subcutaneously for seven alternate days after birth to induce obesity (34). Saline water was injected to the normal control group. Pups were as such kept with mother rats for 21 days. On the 21^st^ day, animals were weaned, housed in standard polypropylene cages, and maintained under normal conditions up to the 42^nd^ day. Body mass index (BMI) and Lee’s index (LI) of rats were determined. Rats with BMI ≥ 20 % and Lee’s indices ≥ 0.3 than the normal control rats were included in the study as obese rats (35). The anthropometric characteristics were observed from the 42^nd^ day to the 70^th^ day, respectively. The model has been established in our lab previously (34). 

***Experimental protocol***

A total of 42 male rats were included and randomly allocated into seven groups, each group consisting of six rats.

Group I/ Normal control: Rat pups treated with 0.9% NaCl (SC) on days 2, 4, 6, 8, 10, 12, and 14 + 0.5% carbo*xy* methyl cellulose (CMC) solution (5 ml/kg, PO) from day 43 to day70. 

Group II / MSG control: Rat pups were given MSG (4 g/kg, SC) on days 2, 4, 6, 8, 10, 12, and 14 + 0.5% CMC solution (5 ml/kg, PO) from day 43 to day 70.

Group III / MSG + Saroglitazar (2 mg/kg): Rat pups were given MSG (4 g/kg, SC) on days 2, 4, 6, 8, 10, 12, and 14 + Saroglitazar (2 mg/kg/day, PO) from day 43 to day 70.

 Group IV / MSG + saroglitazar (4 mg/kg): Rat pups were given MSG (4 g/kg, SC) on days 2, 4, 6, 8, 10, 12, and 14 + saroglitazar (4 mg/kg/day, PO) from day 43 to day 70.

Group V / saroglitazar (4 mg/kg) *per se*: Rat pups treated with 0.9% NaCl (SC) on days 2, 4, 6, 8, 10, 12, and 14 + saroglitazar (4 mg/kg/day, PO) from day 43 to day 70.

Group VI / MSG + fenofibrate (100 mg/kg): Rat pups were given MSG (4 g/kg, SC) on days 2, 4, 6, 8, 10, 12, and 14 + fenofibrate (100 mg/kg/day, PO) from day 43 to day 70.

Group VII / fenofibrate (100 mg/kg) *per se*: Rat pups treated with 0.9% NaCl (SC) on days 2, 4, 6, 8, 10, 12, and 14 + fenofibrate (100 mg/kg/day, PO) from day 43 to day 70.

 Saroglitazar and fenofibrate doses were chosen based on reported literature (26). The drugs were suspended in 0.5% w/v carbo*xy* methyl cellulose (CMC). Daily food intake and weekly body weight were measured. Naso-anal lengths were also measured weekly. Blood samples were collected on the 71^st^ day of life from the tail vein and serum was separated by centrifugation at 3000 rpm for 15 min and frozen at -20 °C. Then animals were euthanized under mild anesthesia. Liver and fat pads (mesenteric, epididymal, and retroperitoneal) were isolated and fixed in 10% formalin solution for histopathological and immunohistochemical studies. 

***Assessment of anthropometric parameters***

Naso-anal lengths and weekly body weight were measured on day 71. The difference between the initial and final weight of animals was monitored for calculation of body weight gain.

BMI was measured from the formula BMI = body weight (g)/length^2^ (cm^2^) (36). Lee’s index (predicts degree of obesity) was calculated from the formula: body weight (g)^1/3 ^/naso-anal length (cm)×1000 (37).

Adiposity index can be measured from the sum of the weights of the epididymal, mesenteric, and retroperitoneal fat deposits/body weight ×100 and was expressed as adiposity percentage (38). The difference between the amount offered and remnants left in the cage cups was used to calculate the food intake and was measured daily at 24 hr intervals. The average food consumption per day was calculated. 

***Measurement of blood glucose***

Blood glucose levels were determined by using the Accu-Check glucometer (Roche Diabetes Care India Pvt. Ltd, Mumbai, Maharashtra, India**)** for blood samples collected from the tail tip. Accu-Check® active test strips (Roche Diabetes Care India Pvt. Ltd, Mumbai, Maharashtra, India) were used for fasting glucose determination.

***Determination of serum insulin and insulin resistance***

Serum insulin levels were measured by using an enzyme-linked immunosorbent assay- ELISA Rat Insulin kit (Krishgen Biosystems, Mumbai, Maharashtra, India) as per the instructions of the manufacturer. 

Insulin resistance was calculated using the formula for the homeostasis model of assessment (HOMA): blood glucose (mg/dl) × serum insulin (IU/ml)/405 (39).

***Assessment of adipocytokines in serum***

Serum levels of leptin, IL-1β, IL-6, and TNFα concentrations were assayed using ELISA kits (Krishgen Biosystems, Mumbai, Maharashtra, India) as per the instructions of the manufacturer.

***Determination of the serum lipids***

Serum levels of triglycerides (TGs), high-density lipoprotein (HDL), and total cholesterol (TC) were measured with commercial kits (Span Diagnostics Ltd., Surat, India) using enzymatic colorimetric assay methods. as per the instructions of the manufacturer. Friedewald’s equation was used to evaluate the serum levels of low-density lipoprotein (LDL) and very-low-density lipoprotein (VLDL): LDL = TC − HDL −VLDL; VLDL = TGs/5 (40).

***Determination of hepatic LDLR***

Low-density lipoprotein levels were determined in liver tissue homogenate using a Rat ELISA LDLR kit (Genxbio Health Sciences, Delhi, India) as per the instructions of the manufacturer. 

***Estimation of malonaldehyde, catalase, and glutathione in liver tissue***

The levels of malonaldehyde (MDA) were estimated according to the method of Okhawa *et al*., 1979 (41). A specified amount of supernatant of tissue homogenate was mixed with the tris-hydrochloride and incubated for 2 hr at room temperature, followed by the addition of 10% trichloroacetic acid. The mixture was centrifuged at 10,000 rpm for 10 min and the supernatant was mixed with the thiobarbituric acid (0.67%) followed by transfer of reacting mixture into boiling water for 10 min and then was brought to the normal temperature. Absorbance was taken at 540 nm and values were expressed as MDA/mg protein (41). The activity of CAT was estimated by the method of Claiborne, 1986 where the weighed amount of PBS and H2O2 was mixed, absorbance was taken at 240 nm, and the result was expressed as nmol H_2_O_2_/min/mg protein (42). Glutathione (GSH) was estimated by the method of Ohkawa *et al*. (1979). The supernatant was mixed with the Tris buffer and DNTB, absorbance was taken at 412 nm and expressed as μmol/mg of protein (43). 

***Histopathological examination***

Isolated tissues of liver and epididymal adipose tissue were fixed in 10% formaldehyde phosphate-buffered saline (PBS, pH = 7.4) and then embedded in paraffin, sectioned, stained with hematoxylin/eosin (H&E) staining, and examined under the Meiji microscope, Japan and sizes of adipocytes were measured using the Image J software (1.52u) Maryland, USA. 

***Immunohistochemical examination of liver tissue***

Immunohistochemical analysis of NLRP3 and NFkB was performed on deparaffinized 4 μm-thick sections of liver tissues and epididymal adipose fat pads. These sections were incubated with fresh 0.3% hydrogen peroxide in methanol for 30 min at room temperature. Briefly, incubation with the antibody against NLRP3 and NFkB was done in deparaffinized tissue slides. 3, 3′diaminobenzidine was used for visualizing the binding sites for antibodies. The sections were then counterstained with hematoxylin, dehydrated using graded alcohols and xylene, and mounted. NLRP3 expression was determined in adipocytes. NFkB expression levels were analyzed by measuring the brown staining of liver tissue by light microscopy (44).

***Statistical analysis***

All values are expressed as mean ± SEM. Significant differences in the body weight, BMI, and Lee’s Index were analyzed by Multivariate analysis of variance (MANOVA) followed by Bonferroni’s *post hoc* test (45). All other parameters were analyzed by one-way ANOVA followed by Tukey’s multiple comparisons test. *P*<0.05 was considered statistically significant. Statistical analysis was carried out using Graph Pad Prism 3.0 software (Graph Pad Software, San Diego, California, USA).

## Results

***In silico interaction of saroglitazar with NLRP3 and NF-*** ***κB***

**Figure 1:**
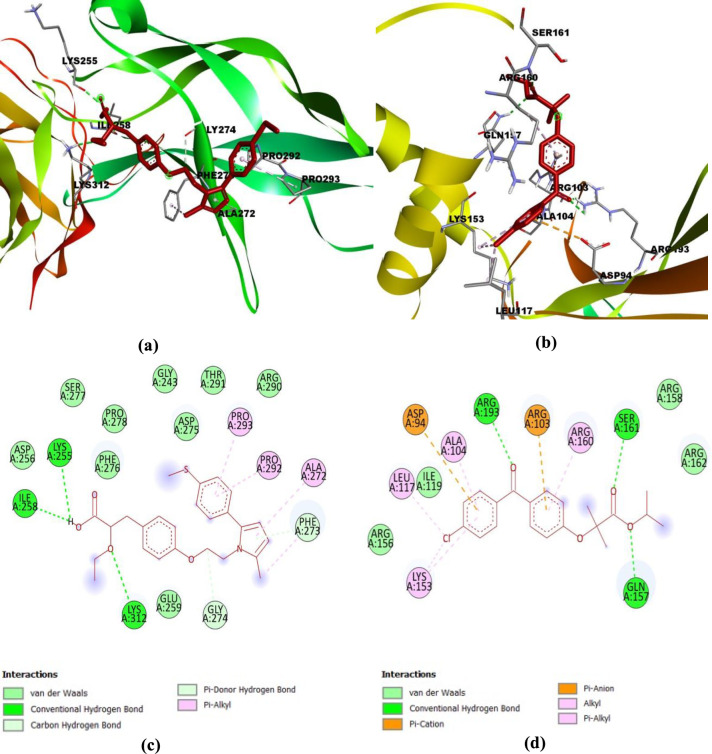
Binding mode and ligand interaction diagram of Saraoglitazar (a), (c) and fenofibrate (b), (d), in the catalytic pocket of NF- κB (PDB ID: 1A3Q)

**Figure 2:**
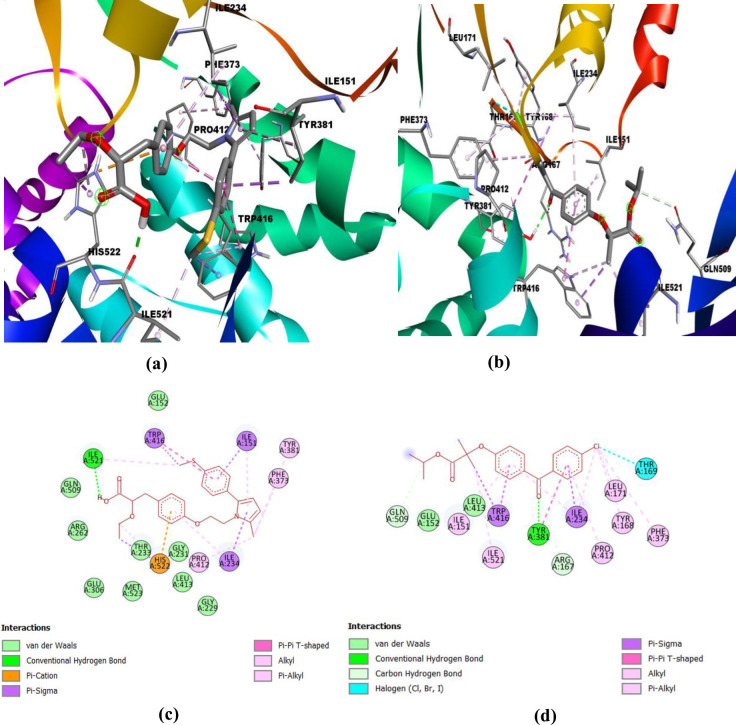
Binding mode and ligand interaction diagram of Saraoglitazar (a), (c) and fenofibrate (b), (d), in the catalytic pocket of NLRP-3 (PDB ID: 7ALV)

Table 1 shows the binding affinity of saroglitazar and fenofibrate with the active binding site of NF- κB (PDB ID: 1A3Q) and NLRP-3 (PDB ID: 7ALV). The molecular docking results depicted that the test drug, saroglitazar (Dock Score: -5.953) displayed significant binding interactions with the ligand-binding domain of NF- κB in comparison with the standard, fenofibrate (Dock Score: -6.911). However, the binding affinity of saroglitazar (Dock Score: -9.111) with NLRP3 was higher than fenofibrate (Dock Score: -8.913). The two-dimensional and three-dimensional interaction diagrams of saroglitazar and fenofibrate with the targets NF- κB and NLRP-3 are shown in Figure 1 and Figure 2, respectively. The hydroxyl group of saroglitazar formed the hydrogen bonds with LYS A:255 and ILE A:258, and an oxygen atom of the ethoxy group formed the hydrogen bond with LYS A:312 of NF- κB. The hydrophobic pocket of NF- κB was enclosed with residues ASP A:256, PHE A:276, PRO A:278, SER A:277, ASP A:275, GLY A:243, THR A:291, and ARG A:290. In the case of NLRP3, the hydroxyl group of saroglitazar formed the hydrogen bond with ILE A:521. One of the aromatic rings showed the pi- pi T-shaped interaction with TRP A:416. GLU A:152, GLN A:509, ARG A 262, GLU A:306, MET A:523, THR A:233, GLY A:231, LEU A:413, GLY A:229 formed the hydrophobic pocket of the active site of NLRP3.

***Effect of saroglitazar on body weight, BMI, and Lee’s index***

**Figure 3:**
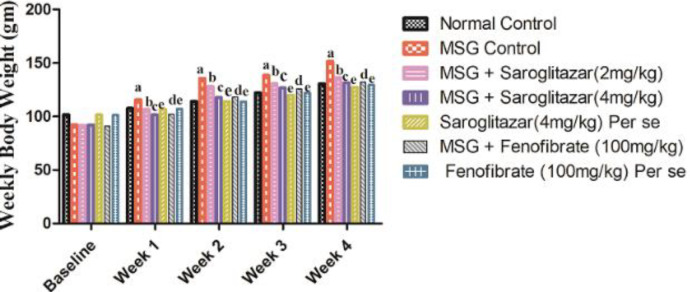
Effect of saroglitazar in MSG-obese Wistar rats on weekly body weight gain

Figure 3 represents the effect of saroglitazar treatment on body weight gain in Wistar rats in various groups. Our results showed that a significant (*P*<0.001) increase in weight gain was observed in MSG-treated rats from the 1st week to the 4th week with normal control rats. Saroglitazar (2 and 4 mg/kg/day, PO) treatment produced a significant (*P*<0.001 ) decrease in body weight gain as compared with the MSG administered group from the 1st week to the 4th week which was comparable with fenofibrate (100 mg/kg/day, PO), that is group VI (*P*<0.001 from the 1st week to the 4th week). Saroglitazar (4 mg/kg/day, PO) significantly (*P*<0.001) decreased body weight gain as compared with saroglitazar (2 mg/kg/day, PO) from the 1st week to the 4th week.

**Figure 4:**
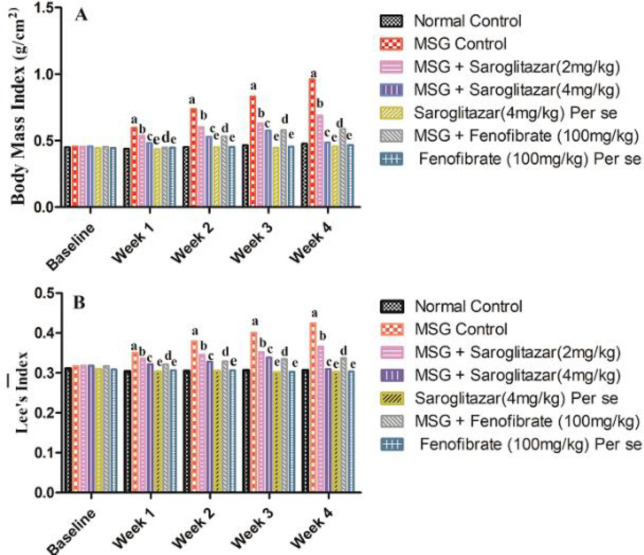
Effect of saroglitazar in MSG-obese Wistar rats on A) BMI and B) Lee’s index

Moreover, BMI and Lee’s index were found to be significantly (*P*<0.001) increased from the 1st week to the 4th week in MSG administered rats in comparison with normal control rats. Administration of saroglitazar (2 and 4 mg/kg/day, PO) produced a significant (*P*<0.001) decrease in BMI and Lee’s index when compared with MSG-treated rats from week 1 to week 4 and the results were comparable with fenofibrate (100 mg/kg/day, PO), that is group VI (*P*<0.001 from the 1st week to the 4th week). Also, administration of saroglitazar (4 mg/kg/day, PO) produced a significant (*P*<0.001) decrease in BMI and Lee’s index as compared with saroglitazar (2 mg/kg/day, PO) from the 1st week to the 4th week. However, no significant (*P*>0.05) difference was found in saroglitazar *per se*, that is group V, fenofibrate *per se*, that is group VII with normal control rats (Figure 4). 

***Effect of saroglitazar on food intake, fat pad weights, and adiposity index***

**Figure 5.**
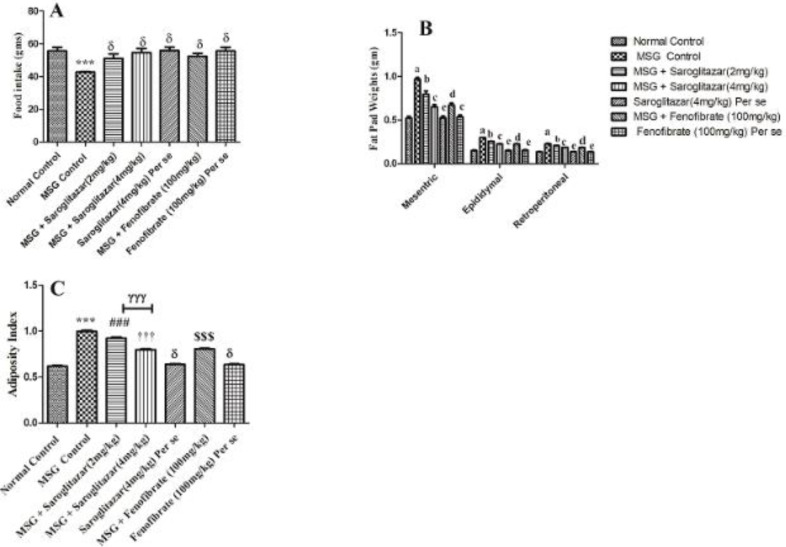
Representative bar diagram showing the effect of saroglitazar in MSG-obese Wistar rats on A) Daily food intake, B) Fat Pad weight, and C) Adiposity index

Figure 5 shows the effects of saroglitazar on intake of food, fat pad weight, and adiposity index in Wistar rats in different groups. It was observed that food intake decreased significantly (*P*<0.001) in MSG administered group compared with normal control rats. Saroglitazar (2 and 4 mg/kg/day, PO) showed a non-significant (*P*>0.05) difference in food intake compared with MSG treated group, that is group II. In addition, fenofibrate (100 mg/kg/day, PO) also showed a non-significant (*P*>0.05) difference in food intake in comparison with the MSG treated group, that is group II. 

Furthermore, there was a significant (*P*<0.001 ) increase in weight of all fat pads (mesenteric, epididymal, and retroperitoneal) in MSG treated group, that is group II, compared with normal control rats, that is group I. Treatment of saroglitazar (2 mg/kg/day, PO), that is group III, showed a significant decrease in fat pad weights (mesenteric, epididymal,) and no significant difference was found in retroperitoneal fat pad weights. On the contrary, saroglitazar (4 mg/kg/day, PO), that is group IV, produced a significant (*P*<0.001) decrease in all fat pad weights which were similar to fenofibrate (100 mg/kg/day, PO), that is group VI. MSG administered rats produced a significant (*P*<0.001) increase in adiposity index compared with normal control rats, that is group I. Saroglitazar treatment (2 and 4 mg/kg/day, PO), that is group III and group IV, produced a significant (*P*<0.001) decrease in adiposity index in comparison with the MSG treated group, that is group II which were comparable with fenofibrate (100 mg/kg/day, PO), that is group VI (*P*<0.001 ). In addition, saroglitazar (4 mg/kg/day, PO) treatment showed a significant (*P*<0.001) decrease in fat pad weights and adiposity index when compared with saroglitazar (2 mg/kg/day, PO) However, no significant (*P*>0.05) difference was found in saroglitazar *per se* (group V), fenofibrate *per se* (group VII), and normal control rats on daily food intake, fat pad weight, and adiposity index in Wistar rats.

***Effect of saroglitazar on the levels of lipids***

 It was found that administration of MSG produced a significant (*P*<0.001) increase in serum TGs, TC, LDL, and VLDL levels and a significant (*P*<0.001) decrease in serum HDL levels when compared with normal control, that is group I. In addition, saroglitazar treatment (2 and 4 mg/kg/day, PO) showed a significant (*P*<0.001) decrease at both doses in serum TC and LDL levels in the MSG treated group. However, saroglitazar treatment (2 mg/kg/day, PO) could significantly reduce TGs (*P*<0.01), and saroglitazar (4 mg/kg/day, PO) produced highly significant (*P*<0.001) reduction in MSG rats. 

 Also, saroglitazar treatment (2 mg/kg/day, PO) could decrease VLDL levels significantly (*P*>0.05) and highly significant (*P*<0.001) decrease was observed in saroglitazar (4 mg/kg/day, PO) in MSG treated rats. The results produced by saroglitazar (4 mg/kg/day, PO) were found to be similar to fenofibrate treatment (100 mg/kg/day, PO), that is group VI (*P*<0.001). Saroglitazar (4 mg/kg/day, PO) produced a significant (*P*<0.001) decrease in serum TGs, TC, LDL, and VLDL levels and a significant (*P*<0.001) increase in serum HDL levels when compared with saroglitazar (2 mg/kg/day, PO) treatment. However, no significant (*P*>0.05) difference was observed in saroglitazar *per se* (4 mg/kg/day, PO), that is group V, fenofibrate *per se*, that is group VII, and the normal control group, that is group I, in Wistar rats (Table 3).

***Effect of saroglitazar on fasting blood glucose, serum insulin, and HOMA-IR***

 MSG treatment in rats produced a significant (*P*<0.001) increase in blood glucose, serum insulin, and HOMA-IR values when compared with normal control rats, that is Group I. Saroglitazar treatment (2 and 4 mg/kg/day, PO), that is group III and group IV, produced a significant decrease in blood glucose at both dose levels. Saroglitazar (2 mg/kg/day, PO) reduced serum insulin levels significantly (*P*<0.01), and significant *P*<0.001) decrease was found with treatment of saroglitazar (4 mg /kg/day, PO).

 Furthermore, significant (*P*<0.001) decrease in HOMA-IR values in saroglitazar (2 and 4 mg/kg/day, PO) treated rats compared with MSG treated rats, that is group II, was observed. The results of saroglitazar (4 mg /kg/day, PO) were comparable with fenofibrate (100 mg/kg/day, PO), that is group VI (*P*<0.001). Also, the observation of saroglitazar (4 mg /kg/day, PO) were found to be significant (*P*<0.001) in comparison with saroglitazar (2 mg/kg/day, PO). However, no significant (*P*>0.05) changes were found in blood glucose levels, serum insulin levels, and HOMA-IR values in saroglitazar *per se *(4 mg/kg/day, PO), that is group V, and fenofibrate *per se* (100 mg/kg/day, PO), that is group VII in comparison with normal control, that is group I in Wistar rats (Table 2).

***Effect of saroglitazar on serum leptin levels***

**Figure 6:**
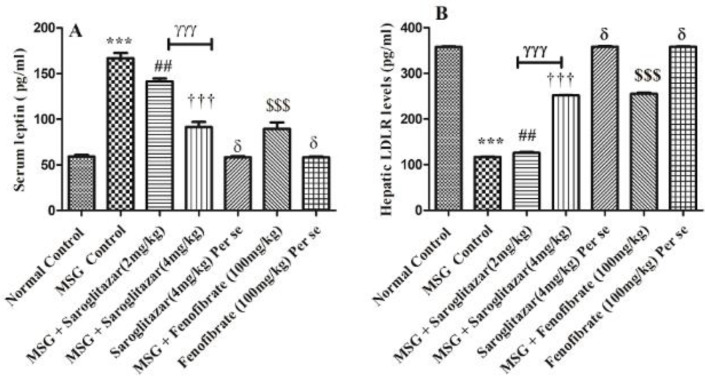
Representative bar diagram showing the effect of saroglitazar in MSG-obese Wistar rats on A) Serum leptin and B) hepatic LDLR protein levels

MSG administration in rats showed a significant (*P*<0.001) increase in serum levels of leptin as compared with normal control rats, that is group I rats. Treatment with saroglitazar (2 mg/kg/day, PO), that is group III produced a significant (*P*<0.01) decrease in serum leptin levels when compared with MSG-treated rats, that is group II. However, highly significant (*P*<0.001) reduction was observed in saroglitazar treatment (4 mg/kg/day, PO) and the results were comparable with fenofibrate (100 mg/kg/day, PO), that is group VI (*P*<0.001). In addition, saroglitazar (4 mg /kg/day, PO) treatment produced significant (*P*<0.001) difference in serum leptin levels with saroglitazar (2 mg/kg/day, PO) treatment. However, no significant (*P*>0.05) difference was observed in saroglitazar *per se* (4 mg/kg/day, PO), that is group V, and fenofibrate *per se* (100 mg/kg/day, PO), that is group VII in comparison with normal control rats (Figure 6). 

***Effect of saroglitazar on serum TNF-α, IL-6, and*** **IL-1β** ***levels***

**Figure 7:**
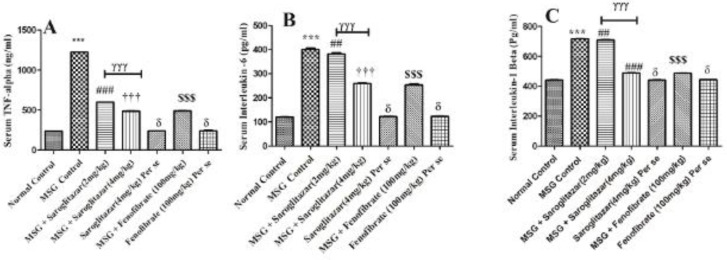
Representative bar diagram showing the effect of saroglitazar in MSG-obese Wistar rats on A) Serum tumor necrosis factor-alpha, B) serum interleukin, and C) Serum interleukin IL-1β

Treatment of rats with MSG significantly (*P*<0.001) increased serum TNF- α, IL-6, and IL-1β levels compared with normal control rats. Saroglitazar (2 and 4 mg/kg/day, PO) treatment, that is group III and group IV showed a significant decrease in TNF- α ( *P*<0.001) levels at both doses with MSG administered rats. Moreover, treatment of saroglitazar (2 mg/kg/day, PO) reduced IL-6 and IL-1β levels significantly (*P*<0.01); however, a highly significant *P*<0.001) decrease in serum levels of IL-6 and IL-1β was observed in saroglitazar (4 mg/kg/day, PO) administered rats when compared with MSG rats and the results of saroglitazar (4 mg/kg/day, PO) were comparable with fenofibrate (100 mg/kg/day, PO), that is group VI (*P*<0.001). In addition, there was a significant (*P*<0.001) difference observed between the saroglitazar (4 mg /kg/day, PO) treated group and saroglitazar (2 mg/kg/day, PO) administered rats. However, no significant difference (*P*>0.05) was found in serum levels of TNF- α, IL-6, and IL-1β in saroglitazar *per se* (4 mg/kg/day, PO), that is group V and fenofibrate *per se* (100 mg/kg/day, PO), that is group VII in comparison to normal control rats, that is group I (Figure 7). 

***Effect of saroglitazar on the levels of LDLR protein***

Administration of MSG produced a significant (*P*<0.001) decrease in LDLR protein levels as compared with normal control rats, that is group I. Treatment of saroglitazar (2 mg/kg/day, PO) showed a significant increase in LDLR protein levels (*P*<0.01) in MSG rats. On the contrary, saroglitazar (4 mg/kg/day, PO) caused a highly significant (*P*<0.001) increase in LDLR protein levels in comparison with MSG treated group which is similar to fenofibrate (100 mg/kg/day, PO), that is group VI (*P*<0.001). Also, the results of saroglitazar (4 mg /kg/day, PO) are significant (*P*<0.001) with saroglitazar (2 mg/kg/day, PO) treated animals. However, no significant difference (*P*>0.05) was observed in LDLR protein levels in saroglitazar *per se* (4 mg/kg/day, PO), that is group V, and fenofibrate *per se* (100 mg/kg/day, PO), that is group VII, and normal control rats, that is group I (Figure 6).

***Effect of saroglitazar on oxidative stress***

The results demonstrated that administration of MSG resulted in significant (*P*<0.001) elevation in the levels of TBARs with normal control rats. According to Table 3, saroglitazar treatment (2 and 4 mg/kg/day, PO), that is groups III and IV, produced a significant (*P*<0.001) decrease in levels of TBARs at both doses and the results were similar to fenofibrate (100 mg/kg/day, PO), that is group V. In addition, a significant (*P*<0.001) difference in the levels of TBARs was observed between saroglitazar (4 mg /kg/day, PO) treated group and saroglitazar (2 mg/kg/day, PO) administered rats. However, no significant difference (*P*>0.05) was observed in TBARs levels in saroglitazar *per se* (4 mg/kg/day, PO), that is group V, and fenofibrate *per se* (100 mg/kg/day, PO), that is group VII, and normal control rats, that is group I (Table 3).

Also, MSG significantly (*P*<0.001) decreased CAT and GSH levels in the liver when compared with normal control rats, that is group I. Saroglitazar treatment (2 and 4 mg/kg/day, PO) showed significant elevation in CAT and GSH levels compared with the MSG treated group, that is group II (*P*<0.001) and the results were similar to fenofibrate (100 mg/kg/day, PO), that is group V. Saroglitazar (4 mg /kg/day, PO) treatment produced significant (*P*<0.001) results in saroglitazar (2 mg/kg/day, Po) administered rats. However, no significant difference (*P*>0.05) was observed in TBARs, CAT, and GSH levels in saroglitazar *per se* (4 mg/kg/day, PO), that is group V, and fenofibrate *per se* (100 mg/kg/day, PO), that is group VII and normal control rats, that is group I (Table 4).

***Histopathological analysis***

**Figure 8:**
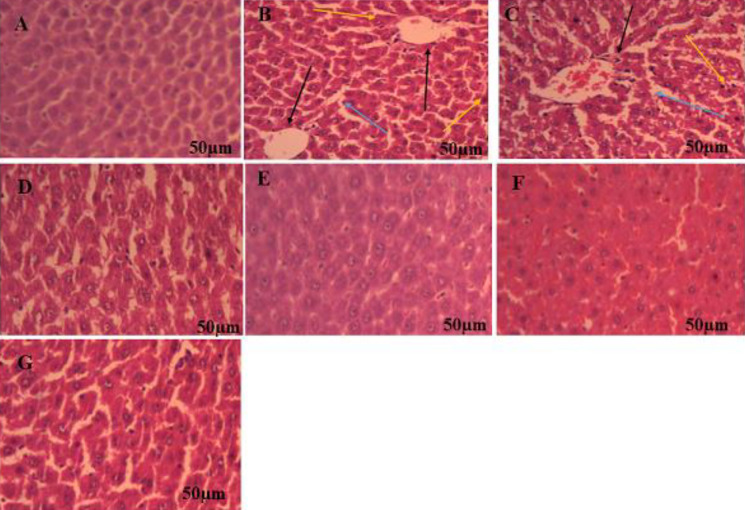
Hematoxylin and eosin (H&E) staining of liver tissue

Histopathological examination by H&E staining of liver tissue showed severe inflammation, vacuole formation, and shifting of the nucleus to one side in MSG-treated rats in comparison with normal control rats wherein, hepatic cells showed normal architecture of hepatic cells (Figure 8). Treatment with saroglitazar (2 mg/kg/day, PO) showed a slight decrease in formation of vacuole, inflammation, and one side nucleus shifting as compared with MSG-treated rats. However, saroglitazar (4 mg/kg/day, PO) decreased inflammation, vacuole formation, and nucleus shifting to one side in comparison with MSG rats which is similar to fenofibrate treatment in MSG administered rats.

**Figure 9:**
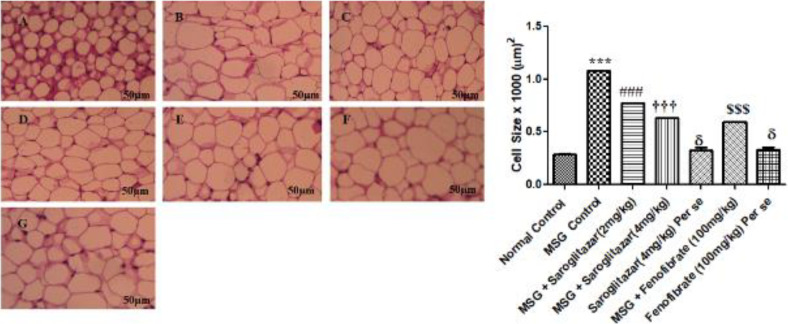
Effect of saroglitazar in MSG-obese Wistar rats on epididymal white adipose tissue mass and size in rats. Photomicrograph showing histopathological changes in adipocyte cell size. The bar indicates 50 µm in the panels (HE: 10X). A) Normal control; B) MSG Control; C) MSG + saroglitazar (2 mg/kg); D) MSG + saroglitazar (4 mg/kg); E) saroglitazar (4 mg/kg) *per se*; F) MSG + Fenofibrate (100 mg/kg); G) Fenofibrate (100 mg/kg) *per se*; and H) The average size of adipocytes in the intraperitoneal adipose tissue. ; valuesValues are mean adipocyte size ± SEM, n = 6 per group. Significance differences were determined by one-way ANOVA followed by Tukey’s Multiple Comparison Test.multiple comparisons test. Adipocyte size was estimated using ImageJ software

Also, histopathological analysis of epididymal adipose fat pads in MSG-administered rats produced a significant increase in the size of adipocytes (*P*<0.001) in comparison with normal control (Figure 9). Both doses of saroglitazar treatment (2 and 4 mg/kg/day, PO) showed a significant (*P*<0.001) reduction in the number and size of adipocytes in MSG rats which are comparable with fenofibrate (100 mg/kg/day, PO) treatment in MSG treated rats. Also, significant results were produced by saroglitazar treatment (4 mg/kg/day, PO) when compared with saroglitazar treatment (2 mg/kg/day, PO). However, no significant difference (*P*>0.05) was observed in adipocyte cell size in saroglitazar *per se* (4 mg/kg/day, PO), fenofibrate *per se* (100 mg/kg/day, PO), and normal control rats (Figure 9).

***Effect on immunohistochemical analysis of liver tissue and epididymal fat depots***

**Figure 10:**
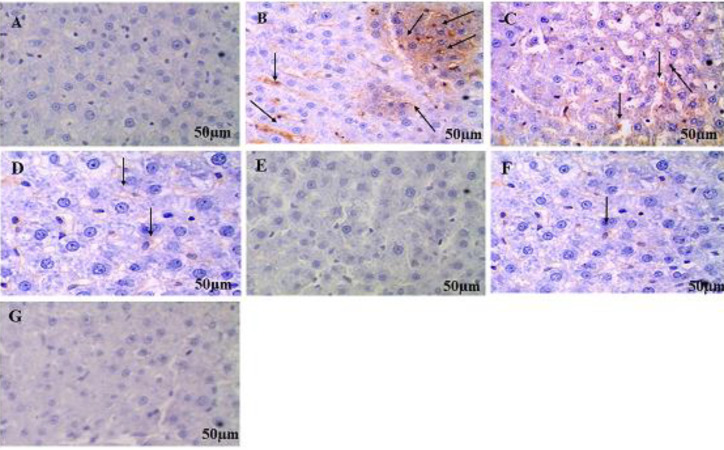
Effect of saroglitazar on nuclear factor kappa-B activation in liver tissue

**Figure 11:**
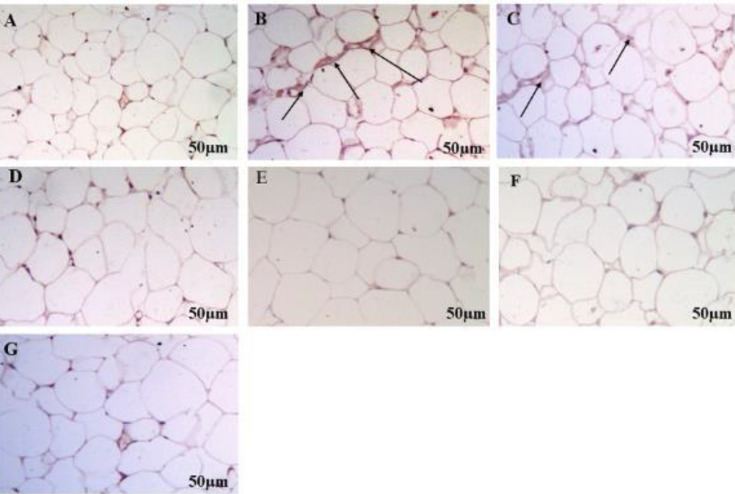
Effect of MSG on NLRP3 activation in epididymal adipocytes

Immunohistochemical staining of normal control rats revealed no positive staining for NF-kB activation in liver cells whereas the MSG-treated rats showed an increase in the number of accumulation of brown-colored immune-stained NF-kB positive cells indicating activation of NFkB pathway revealed by more positive brown staining. Saroglitazar (2 mg/kg/day, PO) produced a mild decrease in NFkB positive cells compared with MSG rats. Saroglitazar (4 mg/kg/day, PO) and fenofibrate treatment (100 mg/kg/day, PO) decreased NFkB positive cells as compared with MSG-treated rats (Figure 10). Immunohistochemical analysis of epididymal fat depots of normal control rats showed no response to NLRP3 inflammasome activation. The MSG-administered rats showed stimulation of NLRP3 inflammasome by the browning of adipocytes. Saroglitazar (2 mg/kg/day, Po) showed a slight decrease in the number of brown adipocytes. However, saroglitazar (4 mg/kg/day, PO) showed a decrease in NLRP3 inflammasome by reduction in browning of adipocytes which was comparable with fenofibrate treatment as compared with MSG-treated rats (Figure 11).

**Table 1:** Dock Score of saroglitazar and fenofibrate with the active binding site of NF- κB (PDB ID: 1A3Q) and NLRP-3 (PDB ID: 7ALV)

**S. No**	**Ligand**	**Dock score**	
		NF- κB (PDB ID: 1A3Q)	NLRP-3 (PDB ID: 7ALV)
1.	Saroglitazar	-5.953	-9.111
2.	Fenofibrate	-6.911	-8.913

Data in the graph are expressed as mean ± standard error of the mean (n = 6 animals per group). Significance was determined by MANOVA followed by the Bonferroni’s *post hoc* test

a *P*<0.001 Normal Control vs MSG Control; b *P*<0.001 MSG Control vs MSG + saroglitazar (2 mg/kg) ; c *P*<0.001 MSG Control vs MSG + saroglitazar (4 mg/kg); d *P*<0.001 MSG Control vs MSG + fenofibrate (100 mg/kg) ; e (*P*>0.05) Normal Control vs saroglitazar (4 mg/kg) *per se* and Fenofibrate (100 mg/kg) *per se*. MSG: monosodium glutamate

Data in the graph are expressed as mean ± standard error of the mean (n = 6 animals per group). Significance was determined by MANOVA followed by Bonferroni’s *post hoc* tests

a *P*<0.001 Normal Control vs MSG Control; b *P*<0.001 MSG Control vs MSG + saroglitazar (2 mg/kg) ; c *P*<0.001 MSG Control vs MSG + saroglitazar (4 mg/kg); d *P*<0.001 MSG Control vs MSG + fenofibrate (100 mg/kg) ; e (*P*>0.05) Normal Control vs saroglitazar (4 mg/kg) *per se*; and fenofibrate (100 mg/kg) *per se*. MSG: monosodium glutamate, BMI: body mass index

Data in the graph are expressed as mean ± standard error of the mean (n = 6 animals per group). Significant differences were determined by one-way ANOVA followed by Tukey’s multiple comparisoncomparisons test

a *P*<0.001 Normal Control vs. MSG Control

b *P*<0.001 MSG Control vs MSG + saroglitazar (2 mg/kg)

c *P*<0.001 MSG Control vs MSG + saroglitazar (4 mg/kg)

d *P*<0.001 MSG Control vs MSG + fenofibrate (100 mg/kg)

e (*P*>0.05) Normal Control vs saroglitazar (4 mg/kg) *per se* and fenofibrate (100 mg/kg) *per se*

***P<0.001 Normal Control vs. MSG Control

###*P*<0.001 MSG Control vs MSG + saroglitazar (2 mg/kg)

††† *P*<0.001 MSG Control vs MSG + saroglitazar (4 mg/kg)

$$$ *P*<0.001 MSG Control vs MSG + fenofibrate (100 mg/kg)

γγγ *P*<0.001 MSG + saroglitazar (2 mg/kg) vs MSG + saroglitazar (4 mg/kg)

δ >0.05 Normal Control vs saroglitazar (4 mg/kg) *per se* and fenofibrate (100 mg/kg) *per se *

MSG: monosodium glutamate, BMI: body mass index

**Table 2:** Effect of saroglitazar on fasting blood glucose, serum insulin, and HOMA-IR in MSG-obese Wistar rats

**Groups**	**Fasting blood glucose (mg/dl)**	**Serum insulin (µIU/L)**	**HOMA-IR**
**Group I / Normal control **	71.67 ± 0.988	7.026 ± 0.166	1.244 ± 0.035
**Group II /MSG control**	139.8 ± 1.400***	11.42 ± 0.492***	3.937 ± 0.146***
**Group III / MSG + Saroglitazar(2 mg/kg)**	133.8 ± 0.477^##^	10.27 ± 0.150^##^	3.394 ± 0.057^###^
**Group IV / MSG + Saroglitazar(4 mg/kg)**	87.17 ± 0.600^†††^	9.099 ± 0.063^†††^	1.958 ± 0.018^†††^
**Group V / Saroglitazar(4 mg/kg) ** ***per*** *** se***	72.17 ± 1.195^ δ^	7.823 ± 0.101^ δ^	1.394 ± 0.027^ δ^
**Group VI / MSG + Fenofibrate (100 mg/kg)**	87.33 ± 0.666^$$$^	8.985 ± 0.046^$$$^	1.938 ± 0.016^$$$^
**Group VII / Fenofibrate (100 mg/kg) ** ***per*** *** se***	72.33 ± 0.881^δ^	6.899 ± 0.132^δ^	1.231 ± 0.019^ δ^

Data are expressed as mean + SEM. Significant differences were determined by one-way ANOVA followed by Tukey’s multiple comparisoncomparisons test MSG: monosodium glutamate. MSG=Monosodium glutamate, HOMA-IR = homeostatic model assessment - insulin resistance

*** *P*<0.001 Normal Control vs. MSG Control

##*P*<0.01 and ###*P*< 0.001 MSG Control vs MSG + saroglitazar (2 mg/kg)

†††*P*<0.001 MSG Control vs MSG + saroglitazar (4 mg/kg)

$$$*P*<0.001 MSG Control vs MSG + fenofibrate (100 mg/kg)

δ>0.05 Normal Control vs saroglitazar (4 mg/kg) *per se* and fenofibrate (100 mg/kg) *per se*

Data are expressed as mean ± SEM (n=6 animals per group). Significance differences were determined by one-way ANOVA followed by Tukey’s Multiple Comparison Testmultiple comparisons test

*** *P*<0.001 Normal Control vs. MSG Control. ## *P*<0.01 and ### *P*<0.001 MSG Control vs MSG + saroglitazar (2 mg/kg). ††† *P*<0.001 MSG Control vs MSG + saroglitazar (4 mg/kg). $$$ *P*<0.001 MSG Control vs MSG + Fenofibrate (100 mg/kg). γγγ *P*<0.001 MSG + saroglitazar (2 mg/kg) vs MSG + saroglitazar (4 mg/kg). δ >0.05 Normal Control vs Saroglitazar (4 mg/kg) *per se* and Fenofibrate (100 mg/kg) *per se*

Data are expressed as mean ± SEM (n=6 animals per group). Significance differences were determined by one-way ANOVA followed by Tukey’s Multiple Comparison Testmultiple comparisons test

*** *P*<0.001 Normal Control vs. MSG Control. ## *P*<0.01 and ### *P*<0.001 MSG Control vs MSG + saroglitazar (2 mg/kg). ††† *P*<0.001 MSG Control vs MSG + saroglitazar (4 mg/kg). $$$ *P*<0.001 MSG Control vs MSG + fenofibrate (100 mg/kg). γγγ *P*<0.001 MSG + saroglitazar (2 mg/kg) vs MSG + saroglitazar (4 mg/kg). δ > 0.05 Normal Control vs saroglitazar (4 mg/kg) *per se*, and fenofibrate (100 mg/kg) *per se*

**Table 3:** Effect of saroglitazar on serum lipid levels in MSG-obese Wistar rats

**Groups**	**TC (mg/dl)**	**TGs (mg/dl)**	**HDL (mg/dl)**	**LDL (mg/dl)**	**VLDL (mg/dl)**
**Group I / Normal control **	51.28 ± 0.595	78.49 ± 0.173	24.08 ± 0.121	11.50 ± 0.678	15.70 ± 0.034
**Group II /MSG control**	99.84 ± 2.919^***^	164.5 ± 0.352^***^	12.67 ± 0.032^***^	55.07 ± 2.699^***^	32.09 ± 0.304^***^
**Group III / MSG + Saroglitazar(2 mg/kg)**	87.21 ± 0.047^###^	159.3 ± 0.414^##^	13.99 ± 0.028^##^	44.71 ± 0.561^###^	31.85 ± 0.082^n.s^
**Group IV / MSG + Saroglitazar(4 mg/kg**	68.36 ± 1.629^†††^	94.65 ± 1.718^†††^	18.76 ± 0.368^†††^	30.67 ± 1.894^†††^	18.93 ± 0.343^†††^
**Group V / Saroglitazar(4 mg/kg) per se**	51.02 ± 0.683^ δ^	78.00 ± 0.243^ δ^	24.08 ± 0.113^ δ^	11.34 ± 0.725^ δ^	15.60 ± 0.048^ δ^
**Group VI / MSG + Fenofibrate (100 mg/kg)**	69.85 ± 2.441^$$$^	93.43 ± 1.738^$$$^	18.74 ± 0.367^$$$^	25.80 ± 2.306^$$$^	18.69 ± 0.347^$$$^
**Group VII / Fenofibrate (100 mg/kg) ** ***per*** *** se***	49.65 ± 0.3946^ δ^	78.03 ± 0.328 ^δ^	23.94 ± 0.206^ δ^	10.09 ± 0.500^ δ^	15.61 ± 0.065^ δ^

Data are expressed as mean ± SEM (n=6 animals per group). Significance differences were determined by one-way ANOVA followed by Tukey’s Multiple Comparison Testmultiple comparisons test

MSG: monosodium glutamate, TC: total cholesterol, TGs: triglycerides, HDL: high density lipoprotein, LDL :low density lipoprotein, VLDL: very low density lipoprotein

***P<0.001 Normal Control vs. MSG Control

##*P*<0.01 and ### *P*<0.001 MSG Control vs MSG + saroglitazar (2 mg/kg)

††† *P*<0.001 MSG Control vs MSG + saroglitazar (4 mg/kg)

$$$ *P*<0.001 MSG Control vs MSG + fenofibrate (100 mg/kg)

δ >0.05 Normal Control vs saroglitazar (4 mg/kg) *per se* and fenofibrate (100 mg/kg) *per se*

**Table 4:** Effect of saroglitazar on thiobarbituric acid reactive substance, glutathione, and catalase in liver tissue in MSG-obese Wistar rats

**Groups**	**TBARS (nmol MDA/mg protein)**	**GSH (µmol of GSH/mg protein)**	**CAT (nmoles of H2 O2 / min/mg protein)**
**Group I / Normal control **	0.3500 ± 0.0044	1.830 ± 0.006	63.41 ± 2.477
**Group II /MSG control**	1.599 ± 0.0157^***^	0.7191± 0.019^***^	23.38 ± 2.953^***^
**Group III / MSG + Saroglitazar(2 mg/kg)**	1.028 ± 0.0071^###^	0.9125 ± 0.006^###^	54.89 ± 3.217^###^
**Group IV / MSG + Saroglitazar(4 mg/kg)**	0.4636 ± 0.010^†††^	1.376 ± 0.034^†††^	59.39 ± 2.365^†††^
**Group V / Saroglitazar(4 mg/kg) ** ***per*** *** se***	0.3466 ± 0.0031^ δ^	1.853 ± 0.009^ δ^	62.46 ± 2.312^ δ^
**Group VI / MSG + Fenofibrate (100 mg/kg)**	0.4539 ± 0.0057^$$$^	1.395 ± 0.028^$$$^	60.26 ± 2.375^$$$^
**Group VII / Fenofibrate (100 mg/kg) ** ***per*** *** se***	0.3439 ± 0.00317^ δ^	1.832 ± 0.025^ δ^	63.82 ± 3.198^ δ^

Data are expressed as mean ± SEM (n=6 animals per group). Significance differences were determined by one-way ANOVA followed by Tukey’s Multiple Comparison Testmultiple comparisons test.

MSG: monosodium glutamate, TC: total cholesterol, TGs: triglycerides, HDL: high density lipoprotein, LDL: low density lipoprotein, VLDL: very low-density lipoprotein

***P<0.001 Normal Control vs. MSG Control

## *P*<0.01 and ### *P*<0.001 MSG Control vs MSG + saroglitazar (2 mg/kg)

†††*P*<0.001 MSG Control vs MSG + saroglitazar (4 mg/kg)

$$$ *P*<0.001 MSG Control vs MSG + fenofibrate (100 mg/kg)

δ > 0.05 Normal Control vs saroglitazar (4 mg/kg) *per se* and fenofibrate (100 mg/kg) *per se*

Photomicrograph showing histopathological changes in hepatic cells. A) Normal Control group; normal histological appearance of liver in control rats. H&E magnification, x 40, B) MSG group, C) MSG + saroglitazar (2 mg/kg), D) MSG + saroglitazar (4 mg/kg), E) saroglitazar (4 mg/kg) *per se*, F) MSG + fenofibrate (100 mg/kg), and G) fenofibrate (100 mg/kg) *per se*. Yellow arrows: one side shifting of nucleus, black arrows: vacuolization, blue arrows: inflammation

***P<0.001 Normal Control vs. MSG Control. ## *P*<0.01 and ### *P*<0.001 MSG Control vs MSG + saroglitazar (2 mg/kg). ††† *P*<0.001 MSG Control vs MSG + saroglitazar (4 mg/kg). $$$ *P*<0.001 MSG Control vs MSG + fenofibrate (100 mg/kg). δ > 0.05 saroglitazar (4 mg/kg) *per se* vs Normal Control, and fenofibrate (100 mg/kg) *per se* vs Normal Control

Photomicrograph showing immunohistochemistry of nuclear factor kappa-B expression in liver cells. A) Normal control; B) MSG Control; C) MSG + saroglitazar (2 mg/kg); D) MSG + saroglitazar (4 mg/kg); E) saroglitazar (4 mg/kg)* per se*; F) MSG + fenofibrate (100 mg/kg); and G) fenofibrate (100 mg/

kg) *per se*; Black arrows are showing nuclear factor kappa-B activation; MSG=Monosodium glutamate

Photomicrograph showing immunohistochemistry of NLRP3 expression in epididymal adipocytes. A) Normal control; B) MSG Control; C) MSG + saroglitazar (2 mg/kg); D) MSG + saroglitazar (4 mg/kg); E) saroglitazar (4 mg/kg) *per se*; F) MSG + Fenofibrate (100 mg/kg); and G) Fenofibrate (100 mg/kg) *per se*; Black arrows are showing NLRP3 activation; MSG: monosodium glutamate

## Discussion

The present study investigated for the first time the ameliorative potential of saroglitazar (2 and 4 mg/kg, PO) in inhibition of adipocyte dysfunction, associated inflammation, and the possible mechanism in MSG obese rats. The key findings of the present study showed that both low and high doses of saroglitazar (2 and 4 mg/kg) inhibited adipose tissue expansion and associated inflammation in a dose-dependent manner. Further, saroglitazar treatment suppressed the inflammatory response by modulation of NLRP3- IL-1β and NF-ƙB pathways; decreased release of various adipocytokines; improved insulin sensitivity; reduced degradation of hepatic LDLR and oxidative stress, thereby, improving impaired serum lipid profile induced by MSG administration in Wistar rats.

Molecular docking predicts the binding affinity, strength, and the most preferable orientation of a drug molecule into the catalytic binding site of the target receptor (46). Therefore, in the current study, molecular docking studies were performed to predict the binding of saroglitazar and fenofibrate on the ligand-binding domain of NLRP3 and NF- κB. The docking study revealed that the binding affinity of saroglitazar (Dock Score: -9.111) with NLRP3 was higher than fenofibrate (Dock Score: -8.913). Moreover, saroglitazar displayed significant binding interactions with the ligand-binding domain of NF- κB comparable with the standard fenofibrate. Therefore, the present study reports that saroglitazar could ameliorate obesity and associated inflammation through involvement of NLRP3 and NF- κB proteins which has not been demonstrated earlier.

BMI measures the body fat content based on weight and height and is highly correlated with body fat stores (47). Lee index is a measure of the degree of obesity and determines the body fat. In the present study, MSG administration showed an increase in body weight, BMI, and Lee’s index along with decrease in food intake. Interestingly, MSG-treated rats also exhibited an increase in fat pad weights and adiposity index. Adiposity index determines the amount of whole-body fat and represents a physical marker in obesity. In addition, MSG increased serum lipid levels including TG, TC, LDL, and VLDL, and decreased levels of HDL. Thus, our results suggest that an increase in body weight, BMI and Lee index resulted due to accumulation of fat rather than food intake. These conditions resulted in adipose tissue expansion, an increase in the size of adipocytes and fat accumulation, hence, inducing obesity. Our results corroborate the findings of Ma *et al*. wherein they have reported that MSG (4 g/kg, SC) administration for 8 consecutive days after birth showed abdominal obesity, impaired lipid levels, insulin resistance, increased lipogenesis, reduced lipolysis in adipose tissues and hypophagia in ICR mice (48). Thus, the results of the present study revealed the development of obesity in MSG-administered rats.

Further, saroglitazar treatment (2 and 4 mg/kg/day, PO) significantly reduced the body weight, BMI, and Lee index in MSG obese Wistar rats indicating the potential of saroglitazar in preventing obesity. In addition, treatment with saroglitazar (2 and 4 mg/kg/day, PO) showed non-significant changes in food intake and decreased the accumulation of fat depots (mesenteric, epididymal, mesenteric, and retroperitoneal) in MSG-obese rats. Furthermore, administration of saroglitazar (2 and 4 mg/kg/day, PO) significantly reduced the levels of TGs, TC, LDL-C, and vLDL and increased HDLc levels in MSG-administered rats. Although, saroglitazar is known to be effective in diabetic dyslipidemia (26), however, the results of our study revealed that saroglitazar possesses the potential to rectify impaired lipid levels associated with obesity in MSG Wistar rats which has not been reported so far.

There is a potential link between obesity and inflammation particularly due to excessive accumulation of white adipose tissue (11, 49). The role of NLRP3 inflammasome and NF-κB in mediating adipocyte dysfunction, inflammation, oxidative stress, and insulin resistance in obesity have been reported in previous studies. Excessive fat mass activates inflammasomes and promotes the release of various pro-inflammatory cytokines IL-1β, IL-6, and TNF-α (15). As a result, in the current study, we evaluated the levels of various pro-inflammatory cytokines in MSG obese rats. The present research indicates that MSG administration resulted in a significant elevation in serum inflammatory cytokines *viz.* IL-1β, IL-6, and TNF-α levels in Wistar rats. However, saroglitazar treatment (2 and 4 mg/kg/day, PO) significantly reduced elevated serum levels of inflammatory cytokines *viz.* IL-1β, IL-6, and TNF-α levels in MSG administered rats. The decrease in the inflammatory state may be attributed to inhibition of NLRP3 inflammasome and NF-κB by saroglitazar treatment in MSG-administered Wistar rats. Our study provides evidence, for the first time that saroglitazar could alleviate obesity and associated inflammation in MSG obese rats by decreasing the production of inflammatory cytokines which play an important role in adipocyte dysfunction. 

Collective evidence suggests that adipose tissue plays an important role in the regulation of lipid and glucose homeostasis (50). The fat deposition is associated with a decrease in glucose uptake by adipocytes which leads to progression of insulin resistance in peripheral tissues. Also, insulin sensitivity decreases with an increase in the size of the adipocytes (51). Moreover, insulin resistance also contributes to reduced glucose transporter (GLUT4) expression in adipose tissue in obesity (52). 

MSG significantly increased levels of blood glucose, insulin, and HOMA-IR values in Wistar rats. Our study is similar to reports of Furuya *et al*. who demonstrated that MSG (2 mg/g body weight) administration from the first to the fifth day after birth increased blood glucose levels and serum insulin levels (52). However, saroglitazar (2 and 4 mg/kg/day, PO) treatment profoundly decreased blood glucose levels, insulin levels, and thereby, HOMA-IR values. Thus, our results indicated that saroglitazar (2 and 4 mg/kg/day, PO) treatment improved insulin sensitivity in adipose tissue, and decreased hyperglycemia and hyperinsulinemia in MSG-obese Wistar rats. In line with our results, previous reports found that high-fat emulsion (HFE) and small doses of lipopolysaccharides (LPS) for 5 weeks in Wistar rats caused adipocyte dysfunction associated with increased blood glucose levels, insulin levels, and insulin resistance in NAFLD which were decreased by saroglitazar (4 mg/kg/day, PO) treatment given from the 3rd week to the 5th week (53). 

The excessive accumulation of fat leads to dysregulation in secretion and metabolism of adipokines, including leptin and insulin which, thereby, develops obesity and associated complications (54). Adipose tissue secretes leptin, an adipokine, which regulates appetite and adiposity and represents the lipid content present in the body (55). The role of leptin resistance in the progression of obesity and inflammation has been reported earlier (48, 56). In the present study, MSG administration caused a remarkable increase in serum leptin levels. This is in line with the previous study of Ma *et al*. who reported that MSG administration causes production of adipokines including leptin in CD-1 mice. Treatment with saroglitazar (2 and 4 mg/kg/day, PO) caused decreased serum leptin levels. Therefore, our results, clearly suggest the role of saroglitazar in the amelioration of hyperleptinemia and leptin resistance which may be one of the potential mechanisms for alleviation of obesity and has not been revealed earlier in the MSG model of obesity. 

Accumulative evidence has revealed the crosslink between inflammation and altered lipoprotein metabolism resulting from dysregulated LDLR by inflammatory cytokines (17, 18). The inflammatory cytokines reduce the hepatic LDLR protein levels causing dyslipidemia which in turn, promotes the accumulation of fat tissues, insulin resistance, and obesity-associated inflammation (57, 58). In our research work, we observed a decrease in hepatic LDLR protein levels in MSG-administered rats. Saroglitazar treatment (2 and 4 mg/kg/day, PO) showed improvement in hepatic LDLR protein levels which indicates that saroglitazar may inhibit hepatic LDLR degradation mediated by MSG, together leading to improvement in lipid homeostasis and amelioration of obesity and obesity-associated systemic inflammation. The role of saroglitazar in improving the hepatic LDLR protein in the amelioration of obesity has not been explored yet. Our study is the first, to provide evidence that saroglitazar could inhibit hepatic LDLR degradation resulting in amelioration of lipid homeostasis, adipose tissue expansion, and associated inflammatory state. 

Previous literature reports the role of oxidative stress and inflammation in the progression of obesity. The liver functions as a key organ for generation of reactive oxygen species (ROS), thereby, promoting fatty acid oxidation, hence, induces an inflammatory response (34). MSG administered rats showed an increase in levels of MDA and decreased GSH and CAT activities in liver tissue. Earlier reports demonstrated that administration of MSG (4 g/kg BW) has been found to induce oxidative stress in hepatic and cardiac tissues in various animal models (34). Saroglitazar treatment produced a decrease in MDA levels and increased GSH and CAT levels in liver tissue of MSG-treated rats. So far, to the best of our knowledge, no study has reported the role of saroglitazar in the inhibition of oxidative stress associated with obesity. 

Histopathological studies by H&E staining of liver tissue showed severe inflammation vacuole formation and shifting of the nucleus to one side in MSG-treated rats. Our findings are corroborated with Bansal *et al*. wherein, they reported that C57BL/6 mice fed with a high-fat diet showed vacuolization and nucleus shifting in hepatic cells (38). Saroglitazar treatment (2 mg/kg/day, PO) showed a slight decrease in inflammation. However, saroglitazar treatment (4 mg/kg/day, PO) significantly decrease the inflammatory changes in hepatic tissue. Adipose tissues normally maintain nutrient stores but an increase in adipocyte cell size causes impaired lipid levels, inflammation and insulin resistance in adipose tissue leading to progression of obesity. Histological analysis of epididymal adipose tissue showed MSG causes hypertrophy of adipocytes. However, saroglitazar treatment (2 and 4 mg/kg/day, PO) showed a significant decrease in hypertrophy of adipocytes in MSG-treated rats. The decrease in hypertrophy of epididymal fat pads enhances insulin sensitivity which is one of the key factors for treating obesity and associated diabetes mellitus (59).

Immunohistochemical studies of liver cells showed no positive staining for activated NF-kB in liver cells of normal control rats. On the contrary, MSG-treated rats showed an increase in the number of immune-stained brown-colored NF-kB positive cells showing activation of NFkB in hepatic cells. Saroglitazar (2 mg/kg/day, PO) produced a slight decrease in NFkB positive cells, however, saroglitazar (4 mg/kg/day, PO) and fenofibrate (100 mg/kg, PO) decreased brown color in comparison with MSG administered rats indicating reduction in activation of NFkB signaling pathway.

Immunohistochemical analysis of epididymal adipose tissue for NLRP3 demonstrated that stimulation of NLRP3 inflammasomes showed browning of adipocytes in MSG-induced obese rats. Previous studies have revealed an increase in the browning of adipose tissue during an inflammatory condition suggesting a potential link between inflammation and browning in adipocytes during obesity (60). Saroglitazar (2 mg/kg/day, PO) produces slightly decreased browning adipocytes, however, saroglitazar (4 mg/kg/day, PO) and fenofibrate (100 mg/kg, PO) decreased brown adipocytes. This indicates that saroglitazar inhibits NLRP3 inflammasomes in MSG Wistar rats.

Lastly, it was observed that saroglitazar treatment at the dose of 4 mg/kg was more effective in significantly decreasing the levels of serum leptin, insulin, IL-6, IL-1β, TGs, HDL, and blood glucose as compared with saroglitazar treatment at the dose of 2 mg/kg in MSG treated Wistar rats. These findings were supported by histopathological and immunohistochemical analysis with saroglitazar treatment at the dose of 4 mg/kg.

Hence, the present study demonstrated that saroglitazar treatment (4 mg/kg/day, PO, for 28 days) effectively ameliorated obesity and associated inflammation, adipokine levels, oxidative stress, and insulin resistance in obesity induced by MSG in Wistar rats. 

## Conclusion

The current study is the first to report the role of saroglitazar treatment in adipose tissue expansion, hypertrophy of adipocytes, and associated inflammation in MSG obese Wistar rats. Saroglitazar treatment ameliorated hyperleptinemia, hyperinsulinemia, insulin resistance, inflammation, oxidative stress, and adipocyte hypertrophy in MSG-treated rats. Saroglitazar ameliorated obesity and associated inflammation via modulation of NLRP3 inflammasome and NF- κB in MSG obese Wistar rats. These results illustrate the antiobesity and antiadipogenic effects of saroglitazar and indicate that it could be a potent therapeutic strategy for treating obesity, associated inflammation, and associated co-morbid conditions.

## Authors’ Contributions

SN, UB, and SHE Designed the study; SN Performed the experiments and collected data; SN, UB, and SHE Contributed to analysis and interpretation of data; SN and UB Drafted the manuscript; SN and UB Revised and edited the article; SN, UB, and SHE Approved the final version of the manuscript. UB and SHE Guided and supervised the research work.

## Conflicts of Interest

No conflicts of interest were declared any of the authors. 
